# Automated Detection of Airway Events in Diverse Hospital Settings: Development and Validation of a Scalable System

**DOI:** 10.1007/s10916-026-02377-2

**Published:** 2026-04-09

**Authors:** Kelly Feldman, Sarah Hamza, Jacqueline C. Stocking, Stephen Murray, Tristan Grogan, Colin J. Sallee, Eilon Gabel, Theodora Wingert

**Affiliations:** 1 Department of Pediatrics, University of California Los Angeles, Los Angeles, USA; 2 Department of Anesthesiology and Perioperative Medicine, University of California Los Angeles, 757 Westwood Plaza, Suite 3304, Los Angeles, USA; 3 Department of Internal Medicine, University of California Davis, Sacramento, USA; 4 Department of Medicine Statistics Core, University of California Los Angeles, Los Angeles, USA

**Keywords:** Algorithms, Information Storage and Retrieval, Clinical Decision Support Systems, Medical Informatics, Health Information Systems

## Abstract

Prolonged invasive mechanical ventilation and extubation failure are common and are associated with increased mortality, length of stay, and costs. Current studies involving airway events usually rely on manual chart review or are dependent on a single data source and typically work because the study populations are limited to homogeneous cohorts with consistent documentation workflow. To support quality improvement and research efforts addressing airway-related outcomes, ways to accurately and reliably identify airway events in electronic health records (EHR) are critical. We developed and validated an automated SQL-based algorithm to obtain high-quality airway event data that performs across diverse clinical settings within a single health system. Using data from all adult and pediatric patients at two large quaternary care hospitals from 2013 to 2025, the algorithm integrated multiple data sources to identify airway events, including ventilator flowsheets, tracheostomy procedures, anesthesia records, admission tables, and lines/drains/airway tables. Data quality was graded using a grading scale that incorporated both the number of data sources and temporal concordance between sources, with higher source count and closer temporal agreement indicating higher data quality (range 1 A [highest] to 3 C [lowest]). Qualitative validation was performed through manual review of 400 records. During the 12-year period, 285,157 unique airway events were identified. Of these, 11.8% were neonatal or pediatric, and 17.5% occurred in critical care or emergency settings. Over 95% of events were graded as high-quality, and this data quality was maintained across diverse intensive care units, perioperative, emergency, and other settings. While external validation is needed, the framework is designed to be adaptable with site-specific validation, and the full codebase is provided to support replication. This automated airway event algorithm generated high-quality airway event data for diverse hospital settings and age-groups within a single health system, and provides a practical framework to improve data quality for airway-related research and quality improvement.

**Supplementary Information:**

The online version contains supplementary material available at 10.1007/s10916-026-02377-2.

## Problem

Invasive mechanical ventilation is associated with a fourfold increase in mortality in adults and is the greatest independent predictor of hospital costs.[1] In pediatric intensive care units (ICU), admissions requiring invasive mechanical ventilation have increased over the past two decades. [2] Significant quality and research efforts are directed at predicting and reducing prolonged ventilation and extubation failure, defined as the need for reintubation after initial extubation [3–10].

For large-scale quality improvement and research efforts related to invasive mechanical ventilation and airway events to be successful, data systems for identifying airway events in electronic health records (EHR) are essential. Existing studies either rely on either manual chart review or utilize single data sources. Studies using single data sources typically are dependent on small homogenous cohorts, such as single-center cardiac ICUs, with uniform documentation and clinical workflows. [11–14] To provide an example, reliance on a single data source may be insufficient in neonatal ICUs and other ICUs where noninvasive mechanical ventilation is common, or in patients who are transferred with endotracheal tubes in place, where the initial placement documentation is often absent. Such methods may also be limited in cases where tracheotomy has been performed or instances of incomplete or variable documentation practices by physicians, respiratory therapists, and nurses.

Currently, there are no standardized, reproducible tools to identify accurately when patients are intubated, extubated, or reintubated across diverse ages and hospital settings. Prediction tools and quality dashboards have significant potential to improve airway-related outcomes; however, their development and successful implementation require high-quality data. Addressing this gap requires scalable, high-quality algorithms capable of accurately identifying airway events in diverse care settings.

Our primary objective was to develop and validate an automated Structured Query Language (SQL)-based algorithm to reliably and accurately identify airway events, including intubation times, extubation times, invasive mechanical ventilation duration, and reintubation events across diverse hospital settings. The secondary objective was to assess the data quality of the algorithm-generated airway data using a custom data quality grading scale.

## Technical Approach

### Data Source & Model Development

**Fig. 1:**
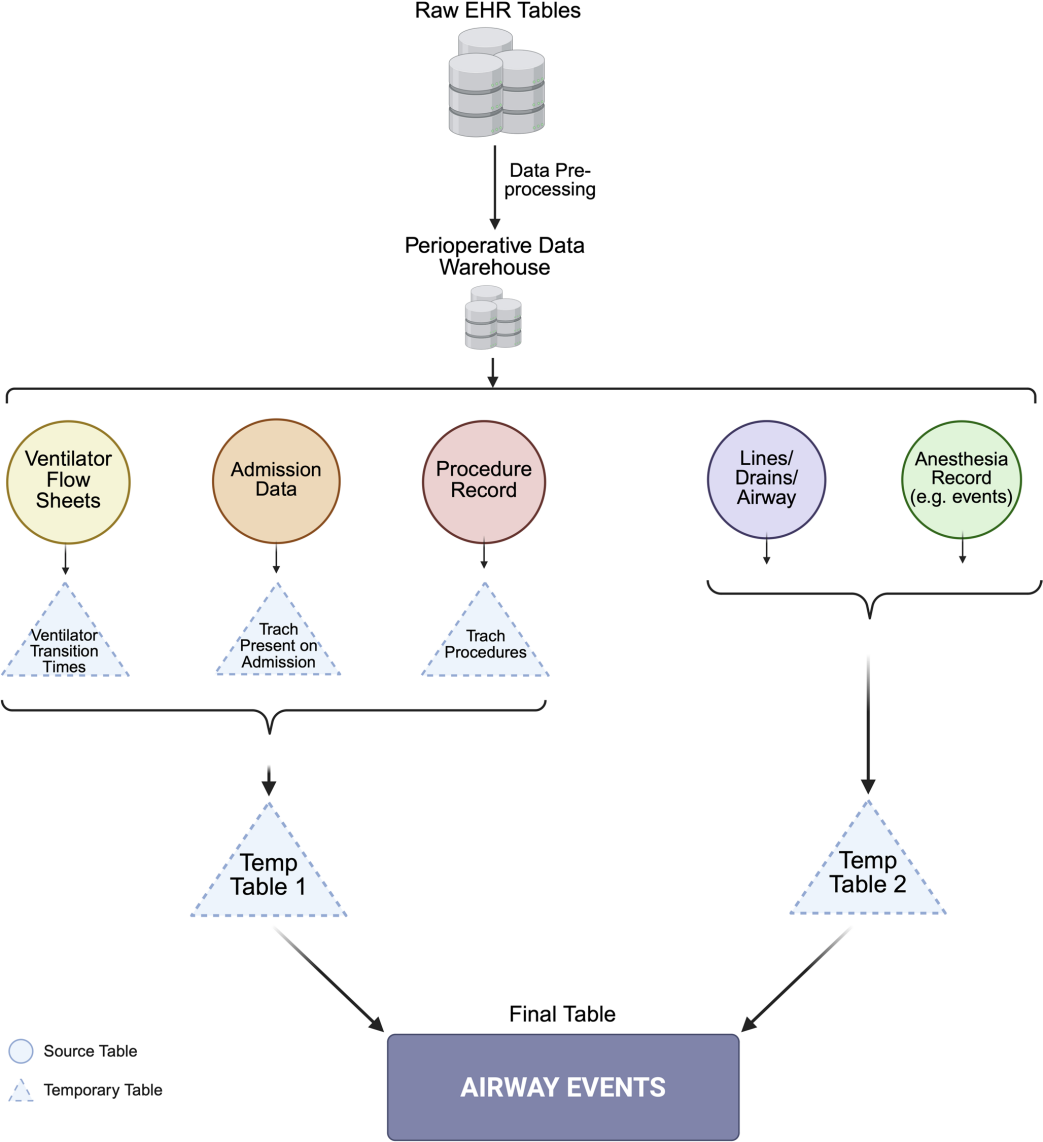
Schematic of Airway Event Algorithm Logic and Sources. This diagram illustrates the conceptual logic of the extraction algorithm, with emphasis on the discrete source tables. Source tables refer to existing tables within Clarity or the Perioperative Data Warehouse that store structured health record data (e.g., patient demographics, encounters, labs). Temporary tables are intermediate datasets created during processing to link or organize data across multiple source tables, facilitating construction of the final analytic dataset. Created in BioRender. https://BioRender.com/rgtavri. Abbreviations: EHR (electronic health record), Trach (tracheostomy), Temp (temporary)

This study was approved by the University of California, Los Angeles (UCLA) Institutional Review Board (IRB #23–000296). Data were extracted from: (1) Clarity, the Microsoft SQL-based relational and reporting database embedded within the Epic EHR system, and (2) the Perioperative Data Warehouse (PDW), a UCLA-developed structured data warehouse optimized for perioperative analytics (Epic Systems Corporation, Verona, WI, USA) [15]. Use of the PDW enabled access to data that had undergone basic labeling, filtering, and categorization, with several key clinical tables already joined. This algorithm is also adaptable for direct extraction from Clarity tables. These sources encompass all patient records from February 2013 through January 2025 from Ronald Reagan Medical Center (RRMC) and Santa Monica Medical Center (SMMC). Airway event data were captured from five EHR sources: ventilator flowsheets, admission data, procedure records, Lines/Drains/Airway (LDA) tables, and anesthesia records. These sources were integrated to identify airway events across diverse clinical settings. A simplified visual schematic of the algorithm’s logic and data sources is provided in Fig. 1, with a more detailed schematic seen in Supplementary Fig. 3. The technical details of the algorithm development can be found in the Supplementary Appendix 1. Also of note, during the 6-month development period, the algorithm output was periodically rerun as the PDW was dynamically updated, with consistent results, providing an additional layer of assurance of the system’s adaptability and consistency over time.

### Validation

Manual review was performed by a single clinician (KF) to assess face validity and characterize scenarios in which the algorithm demonstrated discrepancies compared with chart review. This approach was chosen because the ultimate intended use of the algorithm is to support downstream applications with extubation failure and duration of intubation, where accurate event identification is critical. Cases were randomly sampled from the full cohort and reviewed in sequential batches of 20 to support consistent, high-quality clinician adjudication and minimize reviewer fatigue. A total of 400 cases were reviewed. This number was selected to represent a pragmatic balance between feasibility and obtaining a meaningful qualitative assessment of the 285,157-case dataset. Random sampling across batches ensured representation of a range of algorithm outputs.

### Data Quality Assessment

To assess overall data quality, we developed a data quality grading scale based on two elements:

1. Score (range: 1–3): Score is assigned based on the number of data sources containing the same intubation/extubation event. In this part of the scale, “1” is best, with the greatest number of matching sources, indicating high quality data.
2. Concordance (range: A - C): Concordance is assigned based on the temporal concordance of the recorded times between data sources. In this part of the scale, “A” is best, with data source times within an hour of each other. The lowest quality data concordance, “C”, occurs with sources times more than 12 h apart.

**Fig. 2:**
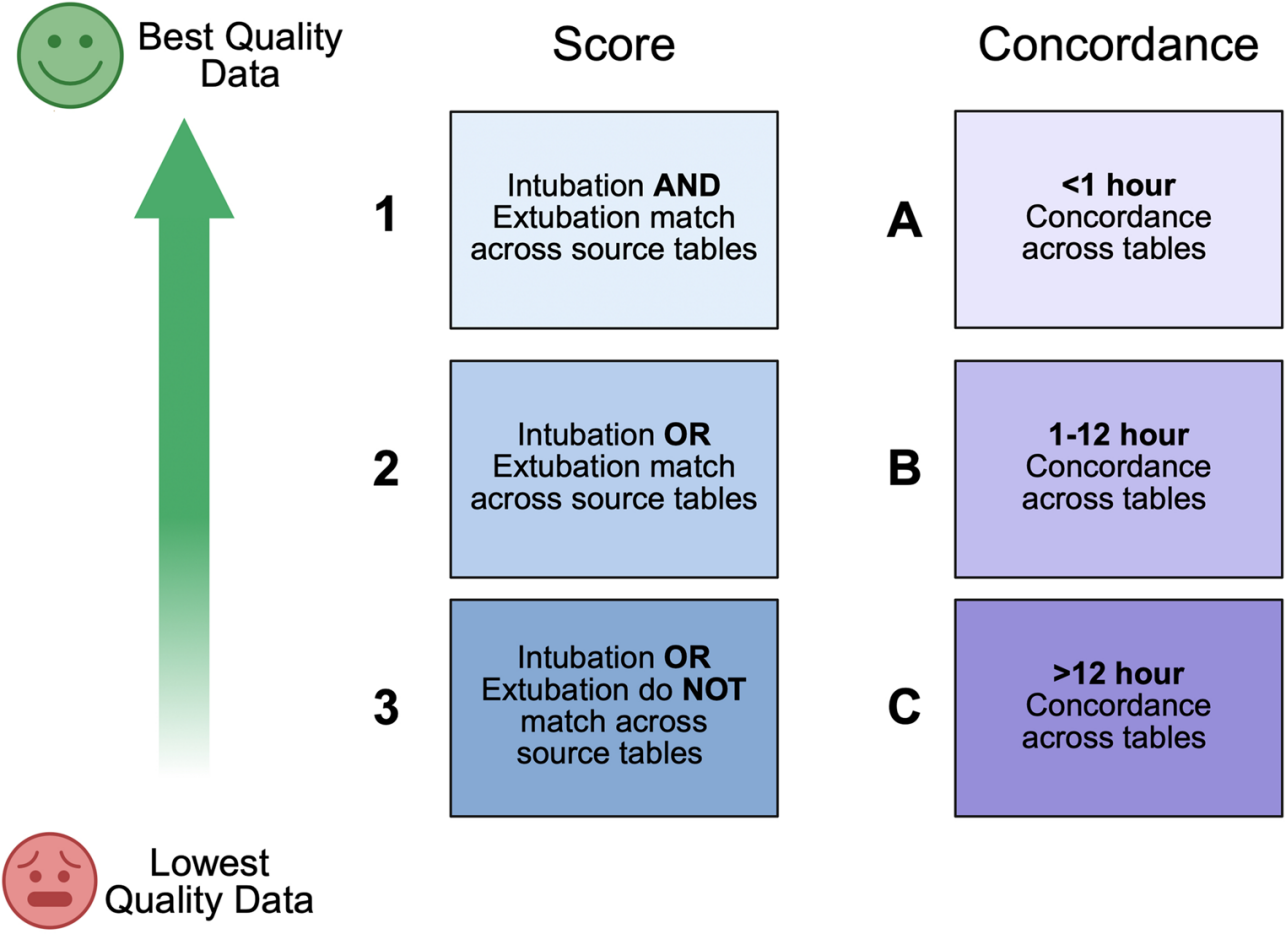
Data Quality Grading Scale. Because no single gold standard exists for this data element, a data quality grading scale was developed. The grade reflects a combination of two components: (1) Source, assigned based on the number of available data sources (with a greater number of sources indicating higher quality data), and (2) Concordance, assigned based on the temporal concordance between data sources (with closer temporal concordance indicating higher quality data.) Data quality grades range from 1 A (highest, best data quality) to 3 C (lowest data quality). Created in BioRender. https://BioRender.com/0435k78

These were combined into a data quality scale (e.g., 1 A = highest data quality, 3 C = lowest data quality), shown in Fig. 2.

## Results

### Data Set

Overall, 285,176 instances of patients being intubated and subsequently extubated at UCLA were examined. Nineteen records (0.007%) containing NULL or invalid data were excluded, resulting in a final dataset of 285,157 records. The algorithm runtime was less than 20 s. Detailed demographics, intubation duration, reintubation rates, and tracheostomy rates are seen in Supplementary Table 1. In the final dataset, 33,679 (11.8%) were pediatric patients (age ≤ 18 years) and 251,478 (88.2%) adults > 18 years of age. The mean patient age was 7.4 years for those ≤ 18 years of age and 55.4 years for those > 18 years of age, for a combined mean of 49.7 years of age. Mean intubation duration was 44.7 h for children and 15.8 for adults, resulting in a combined mean of 19.2 h. Location of both intubation and extubation are found in Supplementary Table 2.

Reintubation is a common research and quality metric that was examined closely. Most patients (98.6%) had no reintubations. Notably, 8,983 (3.2%) patients were reintubated within seven days. Of those patients, 3,777 were reintubated within 48 h of extubation. In total, 57 children and 275 adults (total 332) received new tracheostomies during their ventilation event and 9,205 patients expired. The rate of 48-hour extubation failure in the cohort was 1.8%. Excluding perioperative areas, 1-week reintubation rates range from 4.5 to 11.1% across the different care settings examined.

Extubation location was observed to have different proportions of 48-hour and 2-7-day reintubations (Supplementary Fig. 1). Average intubation duration also varied across locations, ranging from under 50 h in the emergency department to over 200 h in the neonatal ICU and pediatric ICU.

### Data Quality

High data quality (grade 1 A) was observed in 95.6% of records, indicating agreement between a high number of sources and temporal agreement under an hour. High quality data was consistent across hospital locations. Intubation locations showed less consistency in data qulaity compared to extubation locations, but poor data quality (i.e. data quality grade 3 A, 3B, or 3 C) was rare, accounting for < 0.4% of all instances. To further characterize variability, we visualized grading distributions with heat maps (Supplementary Fig. 2), excluding perioperative locations given their predominance (87% of intubations and 82.5% of extubations), which would have skewed results. In both intubation and extubation maps, high data quality (grade 1 A) predominated: for intubation, the “Other” category had the highest grade 1 A frequency, while for extubation, the Cardiac ICU and “Other” categories had the highest grade 1 A frequency. Higher-grade categories (grade ≥1B) appeared infrequently, representing < 19.6% of intubations and < 8.9% of extubations, while poor data quality categories were exceedingly uncommon, occurring in < 2.8% of intubations and < 1.5% of extubations.

### Validation

Manual chart review of 400 cases suggested that the algorithm performed as intended across a range of clinical scenarios. Qualitatively, we observed that instances with poorer temporal concordance occurred primarily in particular clinical scenarios, such as patients transferred from outside hospitals with endotracheal tubes in place, patients who died during hospitalization, or cases with repeated documentation gaps across multiple data sources. In these instances, intubation or extubation times were inferred from admission or discharge timestamps because flow sheet or LDA documentation was unavailable.

## Discussion & Limitations

In this brief report, we describe the development and validation of an automated SQL-based algorithm that reliably identifies airway events (i.e. intubation times, extubation times, intubation duration, and reintubation events) across diverse hospital settings and patient ages. Reintubation rates observed were consistent with other published studies. [16–19] This study addresses a critical gap by providing a potential framework for enabling researchers and quality scientists to obtain high-quality data across diverse clinical settings through a robust informatics approach to capturing airway events.

An important contribution of this study is the conceptual framework of using a data quality grading scale to rigorously evaluate data in the absence of a gold standard. More than 95% of encounters were classified as data quality grade 1 A (highest quality), indicating a high level of robustness of the algorithm across diverse age groups, clinical practice settings, documentation practices, and clinical workflows. While this algorithm was utilized at two hospitals within a single medical system and still requires external validation, it is notable that it achieved high data quality, from neonates to adults and emergency settings to operating rooms. It even performed well in challenging scenarios with tracheostomies, in-hospital death, and clinician documentation inconsistencies. Heat map visualizations of data quality grading have further facilitated rapid identification of high- and low-quality grading distributions, making them suitable tools for quality assurance monitoring in clinical informatics. Use of grading scales like this could potentially be used in other clinical contexts involving large datasets without clear gold standards.

The use of multiple EHR data sources was critical to accurately capture airway events across different clinical and documentation workflows. While LDA tables contain the majority of airway documentation and are commonly used by prior studies, reliance on this source alone resulted in substantial data quality limitations for our institution. Each of the different data sources utilized was helpful in different ways. Ventilator flowsheets were particularly important in neonatal ICU and other populations with frequent use of noninvasive ventilation (e.g. nasal intermittent mandatory ventilation [IMV] or continuous positive airway pressure [CPAP]). Admission and procedure records were critical in identifying patients with existing or newly placed tracheostomies. Anesthesia records were valuable for capturing event timing in cases where LDA documentation was incomplete or missing. Together, these complementary sources generated robustness of the algorithm’s ability to perform across diverse clinical workflows and settings.

The methodology of using an SQL-based approach was chosen to prioritize transparency, reproducibility, and ease of potential implementation within external existing clinical data infrastructures. The novelty of this work lies not in the use of SQL itself, but in the systematic and logical integration and validation of multiple EHR data elements to generate reproducible airway event timestamps from diverse, imperfect sources. While external application will require site-specific adaptation and validation, the underlying logic and framework are designed to be transferable across institutions.

The algorithm’s speed, reproducibility, and ability to generate structured data on intubation, extubation, and reintubation events make it well-suited for future planned retrospective analytics, real-time monitoring, predictive modeling, and integration into dashboards within our tested institution. However we believe with some site-specific adaption, this tool could be very useful to other institutions doing similar research and quality work. Ultimately, shared solutions like this are essential for transforming fragmented EHR records into actionable datasets that support quality improvement, multi-institutional initiatives, and research aimed at reducing ventilator dependence, improving extubation outcomes, and advancing precision care. Indeed, medical informatics is at a pivotal inflection point, with an urgent need for systems that produce high-quality, well-defined data to support research and quality improvement, including applications such as risk prediction models and dashboards [20–25].

## Conclusion

The automated algorithm developed and validated in this study feasibly, accurately, and reliably identified intubation and extubation dates and times across diverse critical care settings at a single health system. Shareable high-quality data systems and definitional standards like this are critical for multicenter data efforts in quality and research. Utilization of automated algorithms like this supports institutional research, quality, and administrative applications, enabling data-driven insights, and may be adaptable for use at external institutions.

## Supplementary Information

Below is the link to the electronic supplementary material.

Supplementary Material 1.

## Data Availability

No datasets were generated or analysed during the current study.
