# Supplementary material for: Automated Detection of Airway Events in Diverse Hospital Settings: Development and Validation of a Scalable System
Source: J Med Syst. 2026 Apr 9;50(1):48. doi: 10.1007/s10916-026-02377-2 (PMC13065591; doi:10.1007/s10916-026-02377-2)
Supplement: Supplementary file 1 — Supplementary Material 1. [file 10916_2026_2377_MOESM1_ESM.docx]

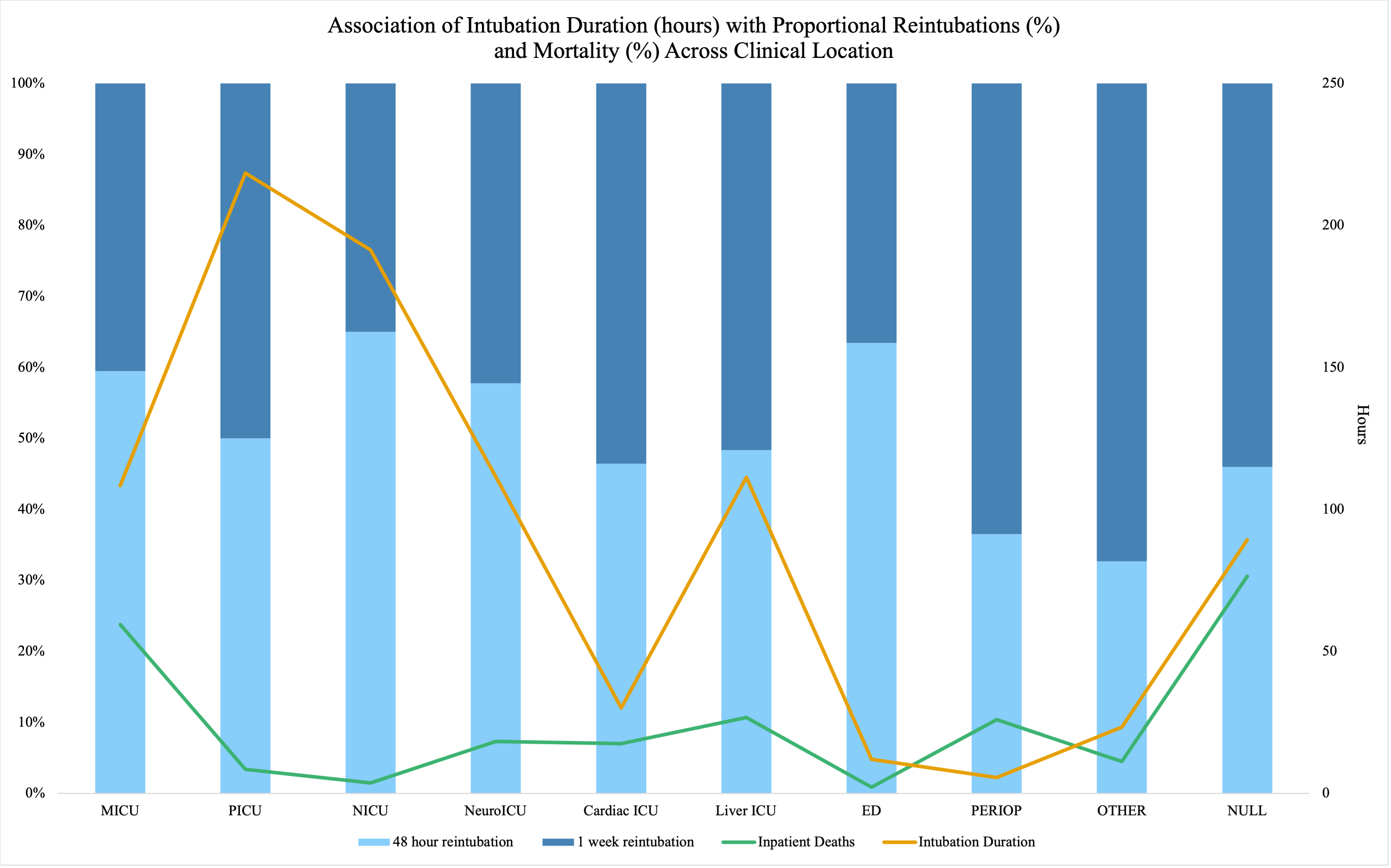
Supplementary Figure 1. Association of Intubation Duration with Reintubations and Mortality across Clinical Location


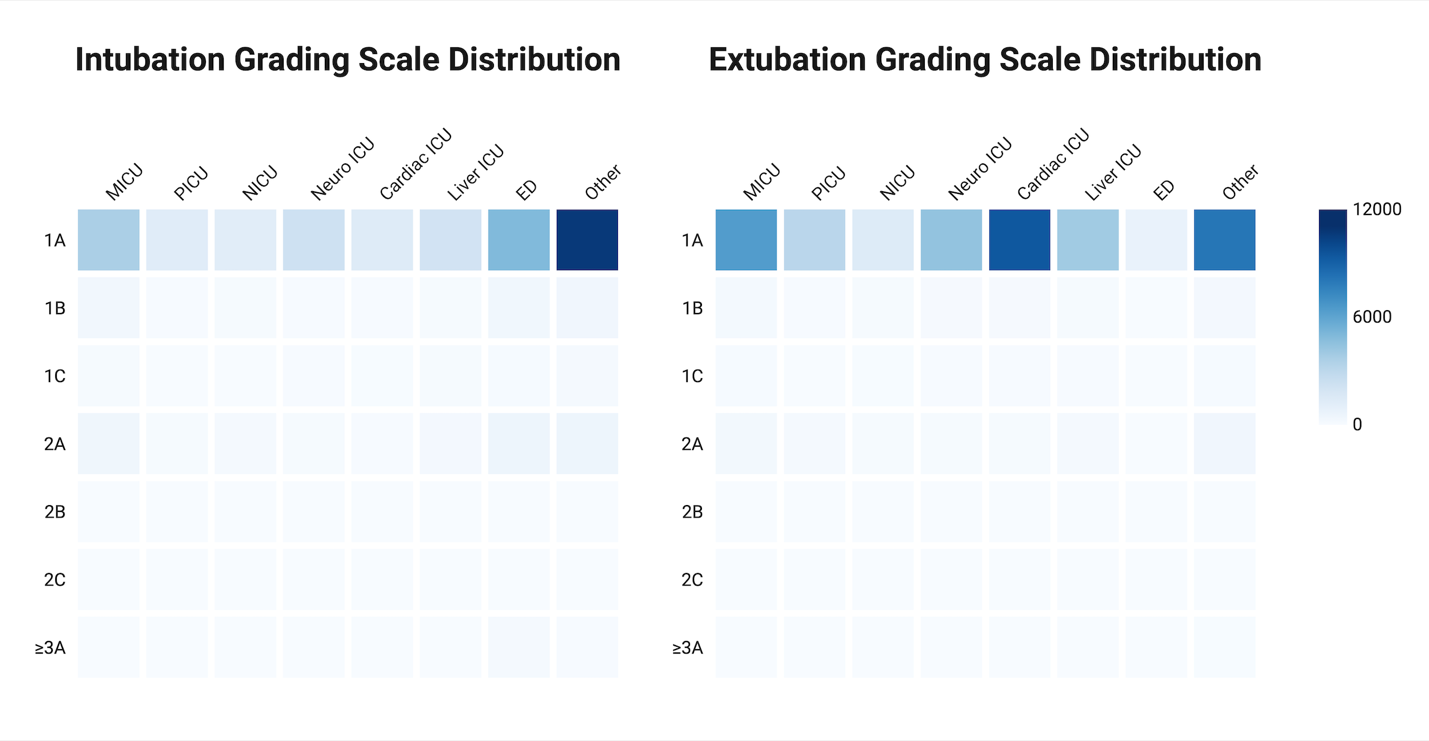
Supplementary Figure 2. Intubation and Extubation Grading Scale Distribution.

Supplementary Figure 3. Detailed Schematic of Algorithm Logic
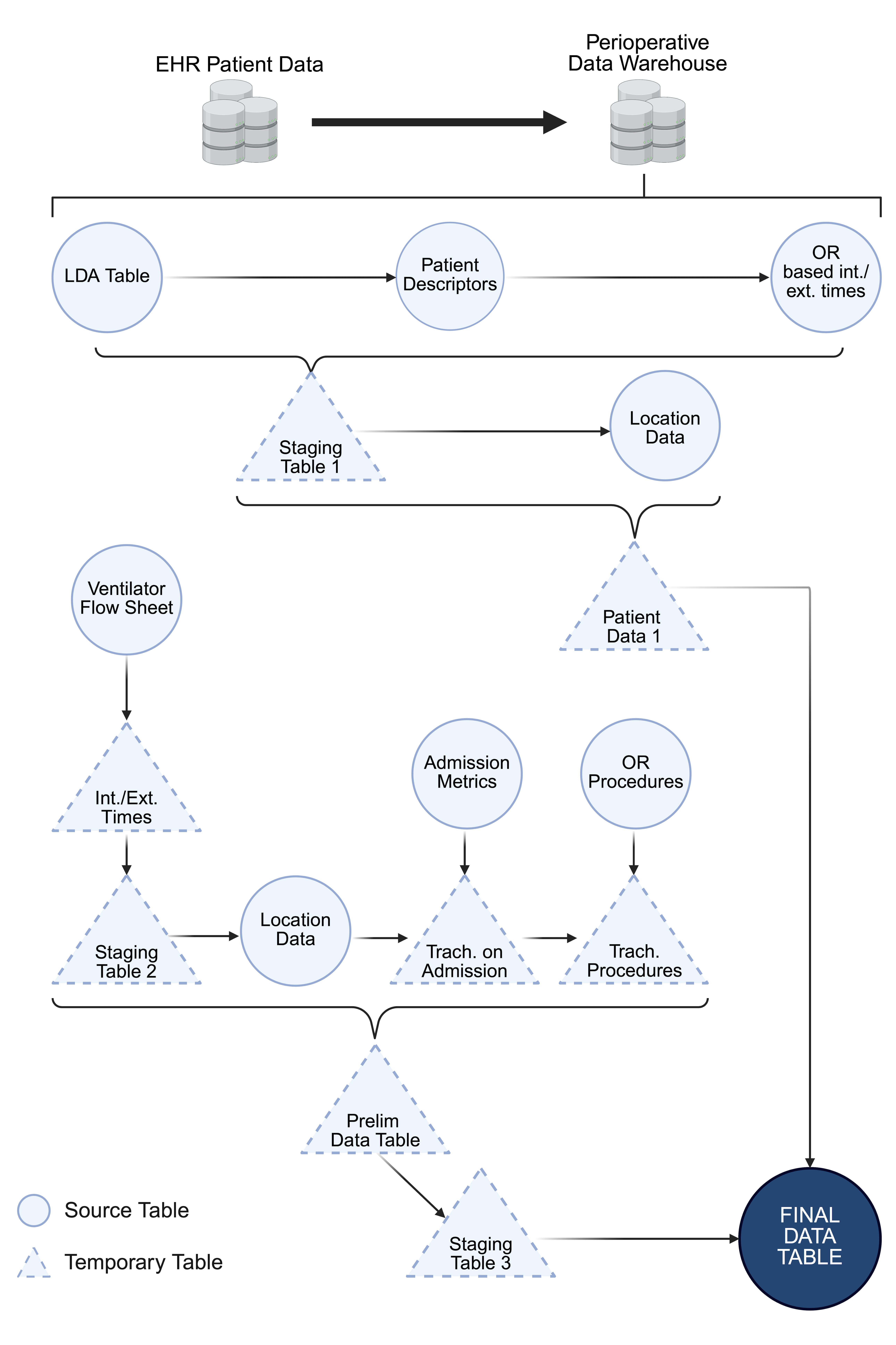


**Supplementary Table 1.** Extraction Algorithm Demographics

|  | **≤ 18 years** | **> 18 years** | **Overall** | ***p*** |
| --- | --- | --- | --- | --- |
| *n (%)* | *33,679* | *251,478* | *285,157* |  |
| **Age (years)** |  |  |  | <0.001 |
| Mean (SD) | 7.4 (6.0) | 55.4 (17.6) | 49.7 (22.7) |  |
| Median (IQR) | 6.0 (2.0, 13.0) | 57.0 (42.0, 69.0) | 54.0 (34.0, 67.0) |  |
| **Intubation Duration (hours)** |  |  |  | <0.001 |
| Mean (SD) | 44.7 (817.0) | 15.8 (234.4) | 19.2 (356.9) |  |
| Median (IQR) | 2.35 (1.3, 5.15) | 2.7 (1.6, 4.7) | 2.7 (1.6, 4.8) |  |
| **Reintubation** |  |  |  | <0.001 |
| No reintubation | 32,428 (96.3%) | 243,746 (96.9%) | 276,174 (96.8%) |  |
| 48-hour | 504 (1.5%) | 3,273 (1.3%) | 3,777 (1.3%) |  |
| 2-7 days | 747 (2.2%) | 4,459 (1.8%) | 5,206 (1.8%) |  |
| **New Tracheostomy** |  |  |  | 0.002 |
| No | 33,622 (99.8%) | 251,203 (99.9%) | 284,825 (99.9%) |  |
| Yes | 57 (0.2%) | 275 (0.1%) | 332 (0.1%) |  |
| **Inpatient Deaths** |  |  |  | <0.001 |
| No | 32,875 (97.6%) | 243,052 (96.6%) | 275,927 (96.8%) |  |
| Yes | 787 (2.3%) | 8,418 (3.3%) | 9,205 (3.2%) |  |
| NULL | 17 (0.1%) | 8 (0.0%) | 25 (0.0%) |  |

|  |  |  | **Extubation** | | | | | | | | | | |
| --- | --- | --- | --- | --- | --- | --- | --- | --- | --- | --- | --- | --- | --- |
|  |  |  | MICU | PICU | NICU | Neuro ICU | Cardiac  ICU | Liver  ICU | ED | PERIOP | OTHER | NULL |  |
|  |  | *n* | *7,615* | *3,684* | *1,850* | *5,134* | *10,534* | *4,742* | *895* | *235,147* | *9,620* | *5,936* |  |
| **Intubations** | MICU | *4,946* | 48.2% |  |  | 0.5% | 0.8% | 1.0% |  | 0.0% | 0.5% | 16.2% |  |
|  | PICU | *1,594* |  | 36.5% | 0.3% |  |  |  |  | 0.0% | 0.1% | 2.1% |  |
|  | NICU | *1,616* |  | 2.6% | 70.6% |  |  |  |  | 0.0% | 0.0% | 2.2% |  |
|  | Neuro ICU | *2,719* | 0.8% | 0.0% |  | 42.8% | 0.2% | 0.7% |  | 0.1% | 0.3% | 2.8% |  |
|  | Cardiac ICU | *1,697* | 0.2% |  |  | 0.3% | 13.6% | 0.4% |  | 0.0% | 0.3% | 1.8% |  |
|  | Liver ICU | *2,710* | 1.1% |  |  | 0.3% | 0.2% | 43.6% |  | 0.0% | 0.2% | 7.0% |  |
|  | ED | *6,636* | 23.1% | 3.0% | 0.1% | 27.9% | 1.3% | 2.9% | 96.0% | 0.3% | 2.1% | 20.4% |  |
|  | PERIOP | *247,934* | 10.8% | 47.8% | 11.7% | 18.0% | 75.0% | 36.8% | 0.2% | 98.3% | 11.4% | 37.9% |  |
|  | OTHER | *12,842* | 8.6% | 3.7% | 1.9% | 3.2% | 5.7% | 6.3% |  | 1.0% | 83.8% | 9.6% |  |
|  | NULL | *2,463* | 7.1% | 6.4% | 15.3% | 7.0% | 3.2% | 8.5% | 3.8% | 0.1% | 1.4% |  |  |
|  | **Total** |  | 100% | 100% | 100% | 100% | 100% | 100% | 100% | 100% | 100% | 100% |  |

**Table 2.** Intubation and Extubation Location as Percentages of Totals by Extubation Locations.

Heat map demonstrating percentages of extubation locations. Sums of percentages do not always add up to 100% due to rounding practices.

#### **Appendix 1.** Algorithm Design and Data Extraction

We designed a multilayered, SQL-based algorithm to identify intubation and extubation times, calculate duration of mechanical ventilation, and detect reintubation events. The algorithm utilized multiple EHR tables cross-referenced using unique patient identifiers (e.g., MRN, patientID, encounterID used as foreign keys) to maximize data completeness and mitigate charting variability. Foreign keys are consistent identifiers that enable accurate linkage of information across different tables, ensuring that data from various sources can be reliably attributed to the correct patient and clinical encounter.

Initial data extraction retrieved key variables, including intubation and extubation times from the Lines, Drains, and Airways (LDA) table, ventilator flowsheets, and perioperative flowsheet documentation. The LDA table, which captures airway type and corresponding placement and removal times, served as the foundation for our multilayered approach. However, due to missing or incomplete values—such as placement times without corresponding removal data—additional sources were queried to fill data gaps and resolve inconsistencies. Other queried tables included those containing admission data, OR case data, charge data, and outcome data.

To identify surrogate indicators of airway transition, we examined shifts in ventilator support modes (e.g., noninvasive to assist control [AC] or synchronized invasive ventilation [SIMV]) – reasoning that transitions in respiratory support documentation could signal intubation or extubation events. Cross-referencing these indicators with LDA data expanded the record count from 298,085 to 340,386. After removing duplicate records, the count decreased to 319,741. Subsequent removal of incomplete records (e.g., records with NULL values in key areas, such as intubation or extubation time. Secondary endpoints with NULL values were allowed to remain.) decreased the record count to 292,014. Finally, implausible records (e.g., intubation durations extending beyond discharge or death), were removed, refining the dataset to 285,157 valid entries.

Patients were assigned a unique ventilation event ID to facilitate information continuity while also allowing for deidentification. Additionally, patients over the age of 90 were further de-identified by adjusting their ages to 90.
